# An Extract of *Glycyrrhiza glabra* (GutGard) Alleviates Symptoms of Functional Dyspepsia: A Randomized, Double-Blind, Placebo-Controlled Study

**DOI:** 10.1155/2012/216970

**Published:** 2011-06-16

**Authors:** Kadur Ramamurthy Raveendra, Jayachandra, Venkatappa Srinivasa, Kadur Raveendra Sushma, Joseph Joshua Allan, Krishnagouda Shankargouda Goudar, Hebbani Nagarajappa Shivaprasad, Kudiganti Venkateshwarlu, Periasamy Geetharani, Gopalakrishna Sushma, Amit Agarwal

**Affiliations:** ^1^Department of Medicine, Srinivasa Diabetic Research Centre, Bangalore 560 050, India; ^2^Department of Medicine, Cambridge Hospital, Bangalore 560 008, India; ^3^Department of Medicine, Manasa Petal Hospital, Bangalore 560 032, India; ^4^Research and Development Centre, Natural Remedies, Bangalore 560 100, India

## Abstract

A randomized, double-blind, placebo-controlled study was conducted to evaluate the efficacy of GutGard, an extract of *Glycyrrhiza glabra*, in patients with functional dyspepsia. The primary outcome variables of the study were the change in the severity symptoms and the global assessment of efficacy. The quality of life was evaluated as a secondary outcome measure. The patients received either placebo or GutGard (75 mg twice daily) for 30 days. Efficacy was evaluated in terms of change in the severity of symptoms (as measured by 7-point Likert scale), the global assessment of efficacy, and the assessment of quality of life using the short-form Nepean Dyspepsia Index. In comparison with placebo, GutGard showed a significant decrease (*P* ≤ .05) in total symptom scores on day 15 and day 30, respectively. Similarly, GutGard showed marked improvement in the global assessment of efficacy in comparison to the placebo. The GutGard group also showed a significant decrease (*P* ≤ .05) in the Nepean dyspepsia index on day 15 and 30, respectively, when compared to placebo. GutGard was generally found to be safe and well-tolerated by all patients. GutGard has shown significant efficacy in the management of functional dyspepsia.

## 1. Introduction

Among various gastrointestinal disorders, functional dyspepsia is one of the most common and costliest clinical conditions in general medical practices. Dyspepsia in the absence of clinically identifiable, structural gastrointestinal lesions is known as functional dyspepsia or nonulcer dyspepsia [1, 2]. The general symptoms of functional dyspepsia include upper abdominal fullness, epigastric pain, belching, bloating, early satiety, nausea, vomiting, regurgitation, heartburn, and loss of appetite [1–5]. 

Though the prognosis remains poorly defined, functional dyspepsia is prevalent worldwide. In Europe and North America, 20% of patients with dyspeptic symptoms had consulted either physicians or hospital specialists; more than 50% patients were on medication often and around 30% of dyspeptics reported taking days off work or schooling [5–7]. Chang reviewed the epidemiology of functional dyspepsia and reported the annual incidence of dyspepsia around 9-10% [8]. Long-term studies indicate that more than 80% of patient populations affected by chronic functional dyspepsia were likely to be persistent after 6-7 years of follow-up [9–11].

Although functional dyspepsia does not seem to be life threatening, the impact remains stressful and leads to huge medical expenses. The dyspeptic patients reported significantly reduced quality of life when compared to general healthy public [8]. As per the published reports, the direct and indirect economic burden due to functional dyspepsia was found to be huge and also has considerable impact on productivity [12]. To achieve a sense of overall well-being, to reduce the cost of treatment and to maintain the quality of life, effective and safe remedies would be a welcome addition for patients with functional dyspepsia. 

Despite the availability of several treatments, the pharmacological interventions were found to be inconclusive and experienced with varied efficacy. With the increasing popularity of medicinal plants globally, many herbal extracts/preparations are evaluated for management of gastrointestinal disorders. The roots and rhizomes of licorice (*Glycyrrhiza glabra *Linn; family: Leguminosae) have been in traditional use for several centuries. The roots of *G. glabra* have expectorant, diuretic, laxative, sedative, antipyretic, antimicrobial, hepatoprotective, antioxidant, and antiadhesive properties [13–16]. In addition, licorice has been reported for enhancing gastric mucus secretion and antiulcer activity [17, 18].

*In vitro *study on glabridin and glabrene (flavonoids present in licorice root) revealed anti-*Helicobacter pylori *(*H. pylori*) activity, and the licorice extract has also shown significant beneficial effect on all forms of *H. pylori* infection [19, 20]. In an earlier *in vivo* study, deglycyrrhizinated licorice (DGL) was found to be effective in alleviation of ulcer in aspirin-induced gastric mucosal damage in rats [21]. The curative effect of DGL in gastric ulcer patients was confirmed during 1970s by clinical trials [22, 23]. Clinically DGL has been used as a main source for the treatment of ulcerative conditions of gastrointestinal disorders like peptic ulcer, canker sores, inflammatory bowel diseases, and so forth [24]. The antiulcer property of licorice extract was also established in gastric ulcer patients. 

Acute oral toxicity study of GutGard, an extract of *G. glabra*, was found to be safe up to 5000 mg/kg in rats. Recently, Chandrasekaran et al. confirmed the dual inhibitory effect of GutGard on derivatives of COX and LOX inflammatory pathways [25]. Specialized licorice extracts have been recently shown to exhibit excellent antiulcer activity in experimental animal models. GutGard has shown marked improvement at different doses (12.5, 25, and 50 mg/kg) in pylorus ligation, cold-restraint stress, and indomethacin induced gastric mucosal injury in albino Wistar rats and the effects were found to be dose dependent [26]. 

From the above considerations, *G. glabra* is found to be an effective agent in the management of several gastroenterological disorders. This study was particularly aimed to assess the efficacy and tolerability of GutGard in patients with functional dyspepsia.

## 2. Materials and Methods

### 2.1. Study Design

The test substance GutGard is a flavonoid rich, root extract of *Glycyrrhiza glabra* developed by Natural Remedies, Bangalore, India. GutGard has following phytochemical specifications, namely, glabridin (≥3.5% w/w), glabrol (≥0.5% w/w), eicosanyl caffeate (≥0.1% w/w), docosyl caffeate (≥0.1% w/w), glycyrrhizin (≤0.5% w/w), and total flavonoids (≥10% w/w). The clinical investigation was conducted over a period of four months (December 2009 to March 2010) as a double-blind, placebo-controlled, randomized manner in two trial centers in Bangalore, India, which included a screening procedure, selection of participants, test medication, and finally posttreatment evaluation. Fifty-four patients were initially diagnosed for functional dyspepsia according to Rome-III criteria [27] and enrolled for the study. Screening was done by physical examination (weight, height, heart rate, systolic and diastolic blood pressure (BP), etc.) and biochemical evaluation before the patients were assigned into the trial. The patients were recruited according to the inclusion and exclusion criteria (Table 1). The purpose and methodology of the clinical trial were explained in simple, understandable language to all the patients. Before randomization, the subjects were asked to completely understand and sign the informed consent form. A copy of informed consent form was issued to trial participants. In addition, investigators clarified queries/doubts of trial subjects if any, prior to signing the consent form. Consent was taken by the investigators of the clinical trial. All the subjects were informed that they can withdraw at any time from participating in the trial without any prior notice. This study was conducted after approval by the Institutional Ethics Committee. 

### 2.2. Randomization and Blinding

**Figure 1:**
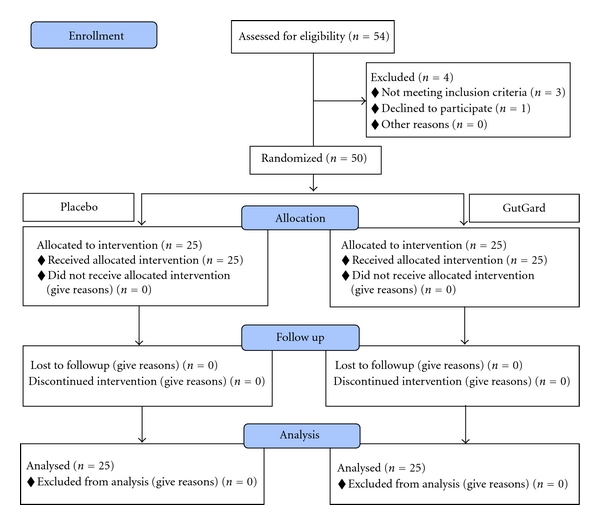
Flow chart of disposition of patients.

After the diagnoses, baseline status was established, four were excluded, and 50 patients were randomly assigned to placebo (*n* = 25) and GutGard (*n* = 25) groups (Figure 1). A list of unique integer random numbers considered as patient code (i.e., random allocation sequence) was generated using a computer-aided programme. As per the random allocation sequence, the containers (either placebo or GutGard capsules) were labeled with unique random numbers. The randomization sequence was developed at Natural Remedies Pvt. Ltd., Bangalore, India, and forwarded to study centre. The entire process was performed in a confidential manner and all the concerned in study centre namely, investigators, patients, and other supportive staff were unaware of the random allocation sequence. The participants fulfilling the selection criteria of the study and after obtaining the written informed consent were enrolled by the study investigators and subsequently the pharmacist dispensed the study medication to the participants taking into consideration the order of enrollment and as per the random allocation sequence. The investigators, patients, and pharmacist dispensing the interventions were all concealed to group assignment. The blinding process was maintained till all the data were compiled and verified for accuracy and then forwarded for statistical analysis. 

Test medication was dispensed by the pharmacist in a container with 30 capsules on day 0 and day 15. The patients were instructed to take placebo or GutGard (75 mg twice daily) with a glass of water after food (one capsule morning and one in the night). The investigational substance was stored as per the recommendation in accessible, controlled area, and pharmacists were accountable for the same. Patients were informed to visit the trial centers on day 15 and day 30 for follow-up. At each visit, the investigators informed the patients to bring the capsule container, and remaining capsule (unused) counts were performed. Patient who took completely the issued capsules was considered to be compliant to the study medication.

### 2.3. Assessment of Efficacy and Tolerability

The primary outcome variables of the study were the change in the severity symptoms and the global assessment of efficacy. A list of 10 gastrointestinal symptoms, namely, upper abdominal fullness, upper abdominal pain, belching, bloating, early satiety, nausea, vomiting, regurgitation, heartburn, and loss of appetite, were considered. The patients were asked to rate themselves for the severity of gastrointestinal symptoms using 7-point Likert scale [28], and the change in severity of symptoms was assessed on days 0, 15, and 30. On day 30, the overall changes in dyspeptic symptoms were calculated, and the measurement was categorized into five grades (symptom free, markedly improved, moderately improved, not changed, and deteriorated) for global assessment of efficacy-an index for the overall response to 30 days of intervention.

The quality of life was evaluated using the short-form Nepean Dyspepsia Index (NDI) as a secondary outcome measure. The NDI is a disease-specific health-related quality of life (HRQOL) instrument consisting of a 10-item questionnaire examining the influence of dyspepsia on five elements (subscales) in patient's health, like tension, interference with daily activities, disruption to regular eating/drinking, knowledge/control over disease symptoms, and interference with work/study. Based on the subscales, the scores of NDI were assessed on days 0, 15, and 30. This total score of NDI gives information on quality of life and impact of illness of dyspeptic patients [29, 30]. The primary and secondary assessment of efficacy was achieved by face to face discussions with trial patients. 

Clinical laboratory investigations were done before and after the interventions in order to assure the safety. The study imposed that medications potentially affecting the gastrointestinal tract were restricted during the trial period.

### 2.4. Data Analysis

The required sample size for difference between two means, that is, for a two-sample *t*-test, was estimated using Snedecor and Cochran formula *n* = 1 + 2*C*  (s/d)^2^ according to Dell et al. [31] as estimated from Holtmann et. al. [1] with *α* value of 0.05 and 1 − *β* = 0.90. Based on this, the required sample size calculated for each arm of GutGard or placebo was 24 subjects or a total of 48 for the complete study. Twenty-five participants from each intervention were considered for the statistical analysis. Characteristics of patients at baseline of two groups were compared by independent samples *t*-test. The change in total symptom scores and Nepean dyspepsia index of each patient on day 15 and day 30 were calculated by subtracting the total symptom scores and Nepean dyspepsia index of day 0 (baseline) from respective observation of each parameters recorded on day 15 and day 30. The total symptoms scores and Nepean dyspepsia index (change from the baseline) of two groups were analyzed by independent samples *t*-test. Effect size, which estimates the change in individual symptom scores relative to the variability in the individual symptoms scores at baseline, was calculated using the following formula.

(1) $$\begin{array}{cc} & \text{Effect}\;\text{size}\\ & \;=\frac{\text{Individual}\;\text{symptoms}\;\text{scores}\;\left(\text{day}\;15\;\text{or}\;30-\text{day}\;0\right)}{\text{Standard}\;\text{deviation}\;\text{at}\;\text{baseline}}. \end{array}$$

A scale with an effect size of 0.8 or larger was considered as magnitude of improvement. The global assessment of efficacy observed in two groups was analyzed by proportion *Z* test [32]. Laboratory investigations recorded on day 0 and day 30 were also analyzed by independent samples *t*-test. The above statistical applications were performed using SPSS software. A two-tailed (alpha = 2) probability value *P* ≤ .05 was considered to be statistically significant.

## 3. Results

Out of fifty-four patients screened for eligibility, three patients were excluded for not meeting inclusion criteria and one patient declined to participate. A total of 50 patients were randomly assigned into two groups, namely, placebo (*n* = 25; 16 males and 9 females) and GutGard (*n* = 25; 15 males and 10 females), and subsequently considered for analysis (Figure 1). 

### 3.1. Demographic Characteristics of Patients

Mean characteristics of treated group versus placebo group at baseline were found to be comparable except for age and diastolic BP in GutGard treated group which were still within normal range (Table 2).

### 3.2. Primary and Secondary Outcome Measures

The change in total symptom scores from baseline of different groups was summarized in Table 3. In comparison with placebo group, GutGard treated group showed a significant decrease (*P* ≤ .05) in total symptom scores on day 15 and day 30, respectively. The effect size of individual symptom scores of different groups was summarized in Table 5. The magnitude of improvement in terms of effect size after 15 and 30 days of treatment was apparently more in GutGard treated group except in “early satiety” as compared with placebo.

With respect to global assessment of efficacy, one patient from GutGard group was completely free from dyspeptic symptoms while none of the patients in placebo group reported symptoms free. Out of 25 patients in each intervention, none in placebo and 14 in GutGard showed marked improvement in symptoms, and the proportion of patients was significantly higher (*P* ≤ .05) in GutGard intervention than that in placebo. Moderate improvement was noticed in nine patients in GutGard treated group and eleven patients in placebo group. The symptoms remained unchanged in fourteen patients of the placebo group while only one in GutGard group and the difference in proportion was significantly less (*P* ≤ .05) in GutGard treated group than the placebo. None of the patients in both groups complained deteriorated condition (Table 4). GutGard supplementation resulted in a significant decrease (*P* ≤ .05) in Nepean dyspepsia index on day 15 and day 30, respectively, versus placebo group (Table 3).

### 3.3. Laboratory Investigations

The blood parameters carried out on day 0 and day 30 in GutGard and placebo groups were within normal limits. Though, there were marginal increase in random blood sugar on days 0 and 30 and decrease in serum creatinine on day 0 in GutGard treated group, these changes were all within the specified normal range (Table 6). There was no study medication-related adverse effect reported during the complete intervention period.

## 4. Discussion

Saad and Chey, in a review on current and emerging therapies for functional dyspepsia, enlisted various approaches employed such as dietary manipulations, modern medicines directed at single or multiple targets within the gastrointestinal and central nervous systems, psychological interventions and of late, and complementary and alternative traditional medicinal systems [11]. Treatment with synthetic medicines, though found to be effective and common, is accompanied with several side effects. In addition, these modern drugs are expensive, alter the normal gastrointestinal functions, and at times may aggravate the existing conditions [24]. 

Use of herbal supplements in the management of gastrointestinal complications, especially for functional dyspepsia, has attracted researchers worldwide. Several herbal formulations have been reported with clinically proven efficacy and safety in the recent past, and screening of medicinal plants for potent antidyspeptic agents appears to be continuing [33]. From the published literature, *G. glabra*, a perennial, temperate zone herb [24], is reported to possess a variety of pharmacological properties such as demulcent [22, 34], anti-inflammatory [18], and antiulcer activities [35] that can be attributed to the beneficial effects of GutGard on gastrointestinal system. A preclinical study on GutGard in albino Wistar rats revealed statistically significant improvements in endpoints, namely, ulcer index, volume, and total acidity of gastric contents in various models of antiulcer activity. Also the study reported potent antioxidant activity with high hydrophilic and lipophilic oxygen radical absorbance capacity (ORAC) value and thereby validated its cytoprotective effect [26].

In the current study, effectiveness of GutGard (75 mg) twice daily for 30 days was evaluated in patients with functional dyspepsia using changes in the pre- and postintervention scores of the study outcome measures. On comparison, GutGard exhibited significant reduction in total symptom scores on day 15 and day 30, marked improvement in global assessment of efficacy, and significantly decreased the Nepean dyspepsia index on day 15 and day 30 than the placebo group. Analysis of effect on individual symptoms of functional dyspepsia has also revealed excellent improvements in GutGard treated group except for early satiety as compared with the placebo group. 

Despite well established, favorable effects of *G. glabra* on digestive system, the available literature indicates the lack of adequate clinical studies on effect of licorice/licorice preparations, as single entity, in functional dyspeptic patients, and connotes the importance of the present study as one of the earliest double-blind, placebo-controlled, clinical trials on efficacy of licorice in the control of functional dyspepsia. 

In the present study, changes in total symptoms scores from baseline values were evaluated, and GutGard supplementation has shown to considerably improve the total symptoms scores. Coon and Ernst, in a review on effects of selected herbal medicinal products in patients with functional dyspepsia, observed that though various techniques were used for measurement of total symptoms scores, few of them were seem to be nonvalidated [33]. Given this consideration, the current study employed the validated 7-point Likert scale reported by van Zanten in *Alimentary Pharmacology and Therapeutics* [28]. Dietary preparations containing licorice as one of the key ingredients have also shown considerable efficacy in patients with functional dyspepsia. A meta-analysis of double-blind, randomized, clinical trials on a polyherbal combination containing licorice (Iberogast) demonstrated excellent overall therapeutic effect in the treatment of functional dyspepsia. The dose and duration of the herbal actives were kept the same in all the individual studies. The findings showed a substantial improvement of symptoms with Iberogast but varying superiority to placebo pertinent to dyspepsia-specific gastrointestinal symptom score [36]. A systematic review on efficacy and tolerability of Iberogast by Melzer et al. also validated the therapeutically related decrease of gastrointestinal symptom-scores in patients with functional dyspepsia [37]. 

Patients' assessments of global efficacy as measured by the proportion of patients without symptoms or with marked improvements have shown the superiority of GutGard treatment (56%) over placebo (0%) and have been found to be in accordance with the changes in the severity of total symptoms. Likewise, the disease-specific quality of life improvements evaluated by NDI also revealed significant advantages resulted by GutGard administration. Although the improvements in quality of life is viewed as a secondary outcome measure in ongoing clinical trials, Talley et al. expressed the prospective use of improvements in the Nepean Dyspepsia Index as a primary objective of treatment in future clinical investigations on gastrointestinal conditions such as functional dyspepsia [29].

With respect to effects on individual symptoms, as evident from effect size, GutGard notably decreased the intensity of symptoms such as upper abdominal fullness and epigastric pain which are considered as important symptoms of functional dyspepsia [38, 39]. Correspondingly, the other parameters of GutGard group also exhibited marked improvements on day 15 and day 30 of the study period except for “early satiety” symptom wherein the effects of the investigational medication and the placebo were found to be comparable. Hence, the overall improvements in total symptoms scores can be attributed to the cumulative and uniform effects of licorice extract on almost all individual symptoms of functional dyspepsia.

On the other hand, relevant gastrointestinal effects (antiulcer activity) of *G. glabra* may provide noteworthy insights in understanding the pharmacological benefits in alleviation of functional dyspepsia. A double-blind clinical study on DGL exhibited ulcer healing properties upon administration of capsules (each with 400 mg actives) for 8 weeks and subjective improvements were recorded in 90% subjects [2]. Lakworthy and Holgate reported antiulcer activity after administration of tablets containing 380 mg DGL in all the 32 patients (100%) endoscopically diagnosed having duodenal ulcer and with chronic history. The beneficial effects were found to be improved as the duration of intervention extended since 56% of the patients recovered after 12 weeks of treatment whereas 78% recuperated after 16 weeks [23]. These findings were in accordance with the current study wherein total symptom scores and NDI were found to be improved over a period of 15 and 30 days. 

As commonly reported in several clinical trials, the present investigation also observed improvements in total symptoms scores of placebo group. However, the improvements were found to be insignificant and not in concurrence with the outcomes of other parameters. In addition to true placebo effect, contribution of spontaneous fluctuations resulting in improvements of symptoms in functional dyspeptic patients is reported in the published clinical studies [3]. Placebo controls are ethically justifiable if usage does not expose research participants to excessive risks of harm [40]. Scientifically, placebo controlled trials require smaller sample size, generate reliable scientific evidence for the evaluation of new substances, and have better internal validity though are less relevant to patient management and have low external validity [41]. 

No treatment-related adverse effects were reported during the study, and GutGard administration was found to be safe and well tolerated by all patients during the complete intervention period. Despite few side effects generally reported with the use of *G. glabra*, patients of the present study did not experience any such side effect that indicates the widely safe nature of the dietary supplement [42]. The available published literature on clinical studies of licorice extracts/formulations also did not report any significant adverse events at various dosage regimens [22, 23]. Isbrucker and Burdock, based on the existing scientific evidence and considering the importance of licorice as a popular food ingredient, reviewed the safety of the medicinal herb and asserted that the current intake levels of licorice products seems to be safe [43].

## 5. Conclusion

The findings of the randomized double-blind, placebo-controlled, clinical trial on GutGard, the root extract of *G. glabra*, revealed significant decrease in symptoms scores in concordance with improvements in almost all individual symptoms and found to be superior to placebo group in the management of functional dyspepsia. The present study also exhibited significantly improved quality of life as evidenced by improved NDI upon administration of the test substance at 75 mg twice daily for 30 days. Hence, GutGard supplementation can be considered as a safe and effective remedy for patients with functional dyspepsia.

## Figures and Tables

**Table 1:** Inclusion and exclusion criteria.

Inclusion criteria	
(i) Diagnosis of functional dyspepsia/nonulcer dyspepsia by fulfilling Rome-III criteria	
(ii) Should be suffering with at least 4 or more symptoms mentioned below and with total symptom score of 20 or more based on 7-point Likert scale	
(a) Upper abdominal fullness	
(b) Upper abdominal pain	
(c) Belching	
(d) Bloating	
(e) Early satiety	
(f) Nausea	
(g) Vomiting	
(h) Regurgitation	
(i) Heartburn	
(j) Loss of appetite	
Exclusion criteria	
(i) Age less than 18 years or over 65 years	
(ii) Advanced chronic illness that would impair follow-up or monitoring	
(iii) Pregnancy or breast feeding	
(iv) Previous surgery for ulcers	
(v) Subjects with previous history of gastroesophageal reflux	
(vi) Subjects with concomitant symptoms of the irritable bowel syndrome	
(vii) Drug and alcohol abuse	
(viii) Mental illness or dementia	

**Table 2:** Characteristics of the patients at baseline (mean ± SE).

Parameters	Placebo (*n* = 25)	GutGard (*n* = 25)
Patients (Male/Female)	16/09	15/10
Age (years)	45.16 ± 2.06	38.12 ± 1.84*
Weight (kg)	67.55 ± 1.75	66.38 ± 2.15
Height (cm)	163.93 ± 1.69	164.29 ± 1.06
Heart rate/min	83.04 ± 1.48	80.32 ± 1.36
BP systolic (mmHg)	128.00 ± 2.10	122.64 ± 1.92
BP diastolic (mmHg)	84.24 ± 0.80	79.92 ± 0.88*
Total symptoms scores of dyspepsia	28.68 ± 0.62	29.96 ± 0.55
Nepean Dyspepsia Index	34.40 ± 1.02	35.64 ± 0.65

*P ≤ .05 versus placebo.

**Table 3:** Efficacy of GutGard on improvement of total symptom scores and Nepean dyspepsia index (mean ± SE).

Groups	Total symptom scores (Change from baseline)		Nepean dyspepsia index (Change from baseline)	
	Day 15	Day 30	Day 15	Day 30
Placebo (*n* = 25)	−5.08 ± 0.57	−8.24 ± 0.76	−4.04 ± 0.49	−6.56 ±0.85
GutGard (*n* = 25)	−11.32 ± 0.77*	−15.20 ± 0.71*	−12.08 ± 0.82*	−19.56 ± 0.85*

*P ≤ .05 versus placebo.

**Table 4:** Effect of GutGard on improvement of global assessment of efficacy.

Groups	Global assessment of efficacy									
	Symptom free	*Z*-value	Markedly improved	*Z*-value	Moderately improved	*Z*-value	Not changed	*Z*-value	Deteriorated	*Z*-value
Placebo (*n* = 25)	0 (0)	—	0 (0)	—	11 (44)	—	14 (56)	—	0 (0)	—
GutGard (*n* = 25)	1 (4)	0.00	14 (56)	4.10*	9 (36)	0.29	1 (4)	3.70*	0 (0)	—

Values in parentheses represent the percentage of patients in each category.

*P ≤ .05 versus placebo.

**Table 5:** Effect of GutGard on the individual symptoms scores.

Parameter	Groups (*n* = 25)	Day 15		Day 30	
		Change in score	Effect size	Change in score	Effect size
Upper abdominal fullness	Placebo	−0.52	0.393	−0.88	0.665
	GutGard	−1.72	5.186	−2.28	6.875
Upper abdominal pain	Placebo	−0.04	0.084	−0.16	0.336
	GutGard	−1.24	1.127	−1.88	1.709
Belching	Placebo	−0.76	0.734	−0.96	0.927
	GutGard	−1.16	2.352	−1.40	2.838
Bloating	Placebo	−0.8	0.755	−1.08	1.019
	GutGard	−1.04	1.733	−1.36	2.267
Early satiety	Placebo	−0.88	0.848	−1.04	1.002
	GutGard	−0.52	0.770	−0.72	1.065
Nausea	Placebo	−0.16	0.225	−0.28	0.393
	GutGard	−0.56	1.065	−0.92	1.749
Vomiting	Placebo	−0.16	0.210	−0.32	0.419
	GutGard	−0.68	1.133	−0.80	1.333
Regurgitation	Placebo	−0.28	0.211	−0.80	0.603
	GutGard	−1.52	1.675	−1.84	2.028
Heartburn	Placebo	−0.84	0.899	−1.44	1.541
	GutGard	−1.52	1.568	−2.12	2.187
Loss of appetite	Placebo	−0.64	0.492	−1.28	0.985
	GutGard	−1.36	0.905	−1.88	1.252

**Table 6:** Results of laboratory blood parameters (mean ± SE).

Parameters	Day 0		Day 30	
	Placebo (*n *= 25)	GutGard (*n *= 25)	Placebo (*n* = 25)	GutGard (*n* = 25)
Haemoglobin(g/dL)	13.30 ± 0.31	13.40 ± 0.33	13.37 ± 0.30	13.58 ± 0.28
Random blood sugar(mg/dL)	86.64 ± 2.76	103.6 ± 2.08*	91.44 ± 1.59	100.72 ± 2.30*
Serum creatinine(mg/dL)	0.93 ± 0.02	0.83 ± 0.03*	0.88 ± 0.02	0.88 ± 0.02
Serum glutamic oxaloacetic transaminase (IU/L)	20.64 ± 1.56	20.50 ± 1.45	20.20 ± 1.08	17.28 ± 1.24
Serum glutamic pyruvic transaminase (U/L)	23.48 ± 1.73	28.03 ± 1.71	20.80 ± 1.07	23.20 ± 1.19

*P ≤ .05 versus placebo.
